# Infective Endocarditis—Impact of Preoperative Neurological Complications on Postoperative Outcome

**DOI:** 10.1111/ene.70539

**Published:** 2026-02-19

**Authors:** George Awad, Bahar Wakeli, Sam Varghese, Boris Kuzmin, Anke Lux, Priya Veluswamy, Mohammad Fadel, Jens Wippermann, Maximilian Philipp Scherner, Max Wacker

**Affiliations:** ^1^ Department of Cardiac and Thoracic Surgery Otto‐von‐Guericke University Magdeburg Magdeburg Germany; ^2^ Institute of Medical Data Science Otto‐von‐Guericke University Magdeburg Germany; ^3^ Department of Cardiac Surgery Medical Faculty and University Hospital, Heinrich‐Heine‐University Medical School Duesseldorf Germany

**Keywords:** cardiac surgery, infective endocarditis, neurological complications, postoperative outcomes, stroke

## Abstract

**Aim:**

Treating infective endocarditis (IE) complicated by neurological events remains challenging and often requires case‐by‐case decisions. Identifying predictors of postoperative complications is key to effective risk assessment and management.

**Methods:**

Data from 191 patients who underwent cardiac surgery for IE were analyzed. Patients were grouped based on the presence or absence of preoperative neurological events (ischemic stroke, TIA, or intracerebral hemorrhage). Univariate and multivariate logistic regression analyses were used to identify predictors of postoperative neurological complications.

**Results:**

Patients with preoperative neurological events underwent surgery later (33 ± 25 vs. 23 ± 23 days, *p* = 0.022), had larger vegetations (1.27 ± 1.88 vs. 0.68 ± 1.08 cm^2^, *p* = 0.029), and more extracranial embolism (55% vs. 10%, *p* < 0.001). Patients with prior neurological complications developed more new cerebral embolic events (65% vs. 3.3%, *p* < 0.001), intracerebral bleeding (20% vs. 1.3%, *p* < 0.001), required longer ventilation (64 ± 89 vs. 59 ± 136 h, *p* = 0.013), and were more frequently discharged to neurological rehabilitation (26% vs. 9%, *p* = 0.008). Preexisting stroke or bleeding significantly increased the risk of cerebral embolism (OR 133.7), prolonged ventilation (OR 2.56), intracerebral bleeding (OR 420), and discharge to neurological rehabilitation (OR 4.71). Independent predictors of new postoperative neurological events were preoperative TIA (OR 19.45), cerebral embolism (OR 10.59), and leukocytosis (OR 8.36). Thirty‐day mortality did not differ between groups (8.3% vs. 7.5%, *p* = 1.0).

**Conclusion:**

Patients with preoperative neurological complications remain at high risk for neurological deterioration in the postoperative course.

## Graphical abstract

In this study of 191 patients with infective endocarditis undergoing cardiac surgery, those who already had neurological complications showed larger vegetations and far more embolic events. They also experienced considerably higher rates of postoperative strokes and intracerebral bleeding, while 30‐day mortality remained similar. Overall, patients with preoperative neurological injury face a clearly higher risk of neurological deterioration after surgery.

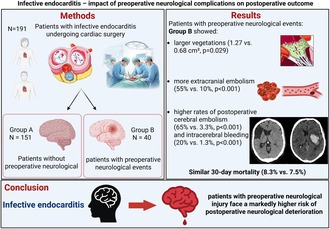

## 1 Introduction

Neurologic events like ischemic stroke, transient ischemic attack (TIA), or intracerebral hemorrhage occur in up to 25% of patients with infective endocarditis (IE) and are considered the main drivers of death or permanent impairment after successful valve surgery [1, 2, 3].

These complications often complicate decision‐making regarding the timing of surgical intervention, as the risks associated with surgery must be carefully balanced against the potential risk for further neurological deterioration under full heparinization necessary during cardiac surgery using the heart‐lung machine.

The current European endocarditis guidelines give detailed recommendations for patients who have had preoperative neurological complications [4]. Treating patients with IE suffering from preoperative neurological complications is often challenged by the variability of clinical presentations and requires a multidisciplinary approach by the endocarditis team, incorporating input from cardiologists, neurologists, infectious disease specialists, and cardiac surgeons, often leading to case‐by‐case decision making [4].

Therefore, identifying risk factors for adverse postoperative outcomes is essential for optimizing treatment strategies and improving the overall prognosis of these high‐risk patients. To address this issue, the present study examines the impact of preoperative neurological events (TIA, ischemic stroke or intracerebral hemorrhage) on the surgical and postoperative outcomes of patients with infective endocarditis in a cohort of 191 patients undergoing cardiac surgery.

## 2 Methods

The data from patients undergoing cardiac surgery for infective endocarditis at the University hospital of Magdeburg between January 2002 and December 2017 were collected retrospectively in Microsoft Excel 2016 (Microsoft Corporation, Redmont, USA). The cohort included 191 patients, who were divided into two groups: Group A (*N* = 151 patients) without preoperative neurologic events and Group B (*N* = 40 patients) with preoperative neurologic events. All patients included in this study were diagnosed with infective endocarditis according to the modified Duke criteria [4], which incorporate clinical, microbiological, and echocardiographic findings. Cases with negative blood cultures were classified based on these criteria using clinical and imaging evidence consistent with IE. Baseline patient data, laboratory and microbiology results, as well as details of the surgery and postoperative outcomes were retrieved from our institutional database. We calculated the EuroSCORE II (European System for Cardiac Operative Risk Evaluation) to predict preoperative mortality. The data were analyzed using SPSS version 24 (IBM Corporation, Armonk, USA). For statistical analyses of baseline and postoperative outcome parameters, contingency tables, the Mann–Whitney *U* test and Chi‐squared test were applied. Univariate logistic regression was used to assess the impact of preoperative cerebral embolism or hemorrhagic transformation on postoperative outcomes. Additionally, a multivariate logistic regression model was applied to identify predictors of postoperative neurological events. For the multivariate logistic regression analysis, all variables with *p* < 0.1 in the univariate analysis were entered into the model.

## 3 Results

The results are represented in tables and therefore not repeated extensively in plain text. Briefly, the patient's baseline characteristics are shown in Table 1. There were no significant differences in patient characteristics, except for the time interval between diagnosis of IE and surgery, where the patients without preoperative neurologic events (Group A) were operated 11 days earlier compared to the group of patients that showed preoperative neurologic events related to IE (group B) (*p* = 0.022). Within Group B, the time interval varied depending on the type of neurologic event. The median waiting time was 6 days for patients with preoperative TIA, 28 days for those with cerebral ischemia, and 56.5 days for those with cerebral hemorrhage. Detailed data are provided in Table S1 regarding the clinical presentation at timepoint of admission, patients in group A did not only exhibit a significant smaller size of vegetation compared to group B (0.7 vs. 1.3 cm^2^, *p* = 0.029), but were also affected less frequently by septic embolism in extracerebral organs (9.9% vs. 55.0%, *p* < 0.001) (Table 2). Interestingly, no differences were found between numbers of patients in critical conditions with the need of catecholamine therapy before operation. Furthermore, the two groups did not differ relevantly in terms of focus for endocarditis or germ spectrum.

**TABLE 1:** Preoperative patient characteristics.

Group A: no preoperative neurologic event; Group B: preoperative neurologic event related to IE (stroke, embolism, hemorrhage, TIA)							
	Total cohort (*N* = 191)	Missing (*N*)	Group A (*N* = 151)	Missing (*N*)	Group B (*N* = 40)	Missing (*N*)	*p*
*Patient characteristics*							
Age (years), mean ± SD	60.8 ± 13.8		61.0 ± 13.0		60.0 ± 16.7		0.944
Female (%)	23		24.5		17.5		0.350
Body mass index, mean ± SD	26.5 ± 4.5		26.7 ± 4.4		25.7 ± 4.9		0.180
Time between diagnosis and surgery (d), mean ± SD	25.2 ± 24.5	39	22.5 ± 22.9	38	33.2 ± 24.5	1	0.022
EuroSCORE II, mean ± SD	33.2 ± 22.3		33.7 ± 22.4		31.5 ± 22.4		0.515
*Past medical history*							
Diabetes mellitus (%)	39.8		39.7		40		0.976
Chronic kidney disease, all stages (%)	37.2		38.4		32.5		0.492
Left ventricular ejection fraction (%), mean ± SD	52.1 ± 11.8	23	51.7 ± 11.4	17	53.9 ± 13.4	6	0.110
Previous cardiac surgery with pericardiotomy (%)	32.5		33.1		30		0.850
Atrial fibrillation (%)	34.6		35.1		32.5		0.759
Chronic obstrutive pulmonary disease (%)	12.6		13.2		10		0.582
Peripherel artery occlusion disease (%)	8.4		9.9		2.5		0.200
Coronary heart disease, all stages (%)	34.7	1	34	1	37.5		0.680
Pulmonary artery hypertonus (%)	14.7		15.9		10		0.349
Current smoker (%)	40	1	41.7		33.3	1	0.340
Arterial hypertonus (%)	70.7		70.9		70		0.915
Hypertlipoproteinemia (%)	30.4		31.1		27.5		0.657
*Laboratory parameters at admission to cardiac surgery*							
Serum creatinine ≥ 104 μmol/L (%)	56.2	13	58.2	10	48.6	3	0.300
Leukocytes ≥ 12.5 Gpt/L (%)	18.2	21	18.7	17	16.7	4	0.784
C‐reactive protein ≥ 20 mg/L (%)	52	66	54.6	54	42.9	12	0.272

**TABLE 2:** Disease pattern related to endocarditis.

Group A: no preoperative neurologic event; Group B: preoperative neurologic event related to IE (stroke, embolism, hemorrhage, TIA)							
	Total cohort (*N* = 191)	Missing (*N*)	Group A (*N* = 151)	Missing (*N*)	Group B (*N* = 40)	Missing (*N*)	*p*
*Clinical presentation*							
Size of vegetation if detectable (area, cm^2^) mean ± SD	0.805 ± 1.308	14	0.679 ± 1.078	12	1.269 ± 1.879	2	0.029
Emergency surgery < 24 h (%)	3.38	43	3.6	40	2.7	3	1.000
Septic embolism, any organ except brain (%)	19.4		9.9		55		< 0.001
Preoperative catecholamines (%)	11.5		11.3		12.5		1.000
*Preoperative neurology related to endocarditis*							
Septic embolism, brain (%)/*N*	14.7		0		70/28		< 0.001
Cerebral hemorrhage (%)/*N*	4.2		0		20/8		< 0.001
TIA (%)/*N*	2.1		0		10/4		0.002
*Focus*							
Valve is primary focus (%)	74.9		77.5		74.2		0.690
*Secondary focus*							
Throat/ear/nose (%)	3.1		1.3		10		0.018
Dents (%)	14.1		15.2		10		0.458
Bone (%)	0.5		0.7		0		1.000
Extremities (%)	3.1		3.3		2.5		1.000
Wounds (%)	1.0		1.3		0		1.000
Vascular access (%)	0.5		0.7		0		1.000
Cholecystitis (%)	0.5		0.7		0		1.000
Drug abuse (%)	0.5		0.7		0		1.000
Prostate biopsy (%)	1.0		1.3		0		1.000
Port (%)	0.5		0.7		0		1.000
*Germ spectrum in blood cultures*							
Stapylococcus species (%)	33		30.5		42.5		0.186
Steptococcus species (%)	16.8		17.2		15		0.817
Enterococcus species (%)	11.5		13.2		5		0.175
Negative (%)	32.5		33.1		30		0.850

Regarding the operative data, no significant differences were detected between group A and group B (Table 3).

**TABLE 3:** Operative data.

Group A: no preoperative neurologic event; Group B: preoperative neurologic event related to IE (stroke, embolism, hemorrhage, TIA)				
	Total cohort (*N* = 191)	Group A (*N* = 151)	Group B (*N* = 40)	*p*
Cardiopulmonary bypass time (min), mean ± SD	135.7 ± 75.5	139.2 ± 81.2	122.7 ± 46.4	0.412
Aortic cross clamp time (min), mean ± SD	95.0 ± 51.0	96.8 ± 53.8	88.4 ± 38.3	0.470
ECMO or IABP intraoperatively (%)	1.047	1.325	0	1.000
*Affected valve*				
Aortic valve, singular (%)	49.2	49.7	47.5	0.860
Mitral valve, singular (%)	30.4	28.5	37.5	0.334
Tricuspid valve, singular (%)	2.1	2.6	0.0	0.581
Aortic and Mitral valve (%)	15.2	15.2	15	1.000
Valve replacement, biological (%)	34.6	32.5	42.5	0.235
Valve replacement, mechanical (%)	62.3	64.2	55.0	0.284
Only valve repair (%)	3.1	3.3	2.5	1.000
Concomitant CABG (%)	27.7	29.1	22.5	0.436
Combination with reconstruction of other non IE affected valve (%)	17.8	20.5	7.5	0.064

While Euroscore II predicted a mortality of 33% for the overall patient cohort (Table 1), the true 30‐day mortality in the overal patient cohort was 8.4% (Table 4).

**TABLE 4:** Postoperative course.

Group A: no preoperative neurologic event; Group B: preoperative neurologic event related to IE (Stroke, Embolism, Hemorrhage, TIA)							
	Total cohort (*N* = 191)	Missing (*N*)	Group A (*N* = 151)	Missing (*N*)	Group B (*N* = 40)	Missing (*N*)	*p*
Operative mortality (within 72 h) (%)	3.7		4.6		0		0.348
Mortality within 30 days (%)	8.4		8.3		7.5		1.000
New cerebral embolism (verified by CT scan) (%)	16.2		3.3		65		< 0.001
New cerebral hemorrhagic transformation (verified by CT scan) (%)	5.2		1.3		20		< 0.001
Mean ventilation time (h), mean ± SD	60.2 ± 127.7	1	59.3 ± 136.2		63.7 ± 88.6	1	0.013
Ventilation > 24 h	28.4	1	25.2		41	1	0.050
Tracheotomy (%)	6.8		3		10		0.477
Reexploration (%)	7.9		7.3		10		0.741
Need for permanent pacemaker implantation (%)	15.7		16.6		12.5		0.531
Interval between operation and discharge (days), mean ± SD	15.6 ± 10.2		15.7 ± 10.4		14.9 ± 9.5		0.681
ICU stay length (days), mean ± SD	7.6 ± 8.4		7.7 ± 9.1		7.2 ± 5.5		0.320
Discharge to neurological rehabilitation (%)	12.5	15	8.8	14	25.6	1	0.008
*Detection of microbacteria in resected valve material (%)*							
Negative	80	1	78.7	1	85		0.374
Staphyloccocus species (% of detected bacteria)	6.8		6		10		0.477
Streptococcus species (% of detected bacteria)	2.1		2		2.5		1.000
Enterococcus species (% of detected bacteria)	6.8		8.6		0		0.074

While the mortality was not different between the two groups, there were further significant differences in the postoperative course between the two groups (Table 4). First, patients from group B suffered significantly more often from new cerebral complications such as bleeding or embolism (*p* < 0.001), and second, the patients from group B were significantly longer under mechanical ventilation (59.3 vs. 63.7 h, *p* = 0.013). Finally, patients in Group B were more frequently transferred to neurological rehabilitation (8.8% vs. 25.6%, *p* = 0.008).

In an univariate logistic regression model, patients with preoperative cerebral embolism had an extremely high risk for a new cerebral embolism in the postoperative course (OR 133.71, *p* < 0.001); however, the odds for 30‐days mortality and postoperative cerebral bleeding were not increased (Table 5). Despite this, patients with preoperative cerebral embolism had a 2.6 times higher risk for a ventilation time > 24 h (*p* = 0.025), but interestingly, the risk for tracheotomy was not increased.

**TABLE 5:** Impact of preoperative cerebral embolism on postoperative outcome parameters.

Impact of preoperative cerebral embolism (*N* = 28) on postoperative outcome parameters			
	OR	95% CI	*p*
Death (30 days)	0.82	0.18–3.82	0.799
New cerebral embolism (verified by CT scan)	133.71	36.39–491.36	< 0.001
New cerebral hemorrhagic transformation (verified by CT scan)	0.63	0.078–5.21	0.671
Renal failure ≥ AKIN Stage 2	0.20	0.03–1.28	0.089
Ventilation time > 24 h	2.56	1.12–5.82	0.025
Tracheotomy	2.852	0.81–9.99	0.101
Operative reexploration of any kind	0.89	0.19–4.16	0.880
Discharge to neurological rehabilitation	1.77	0.59–5.27	0.309

While the risk for discharge to neurological rehabilitation was not increased for patients with preoperative cerebral embolism, it was indeed 4.7 times higher for patients with preoperative cerebral bleeding (*p* = 0.044) (Table 6). These patients were also at high risk for a new cerebral bleeding episode during the postoperative course (OR 420.0, *p* < 0.001).

**TABLE 6:** Impact of preoperative cerebral hemorrhage on postoperative outcome parameters.

Impact of preoperative cerebral hemorrhage (*N* = 8) on postoperative outcome parameters			
	OR	95% CI	*p*
Death (30 days)	1.60	0.18–13.89	0.670
New cerebral embolism (verified by CT scan)	0.73	0.09–6.14	0.771
New cerebral hemorrhagic transformation (verified by CT scan)	420.00	38.65–5464.48	**< 0.001**
Ventilation time > 24 h	1.01	0.19–5.36	0.993
Operative reexploration of any kind	1.72	0.20–15.03	0.622
Discharge to neurological rehabilitation	4.71	1.04–21.28	0.044

In a multivariate logistic regression model, preoperative TIA (*p* = 0.046), preoperative cerebral embolism (*p* = 0.021) and preoperative leukocyte count > 12.5 Gpt/L (*p* = 0.019), but not preoperative cerebral bleeding (*p* = 0.215) were identified as predictors of postoperative neurologic events (Table 7).

**TABLE 7:** Multivariate logistic regression model of predictors of postoperative neurologic events (embolism or hemorrhage or TIA).

Multivariate logistic regression model of predictors of postoperative neurologic events (embolism or hemorrhage or TIA)			
Variable	OR	95% CI	*p*
Preoperative TIA (*N* = 4)	19.45	1.05–360.80	0.046
Preoperative cerebral embolism (verified by CT scan)	10.59	1.43–78.46	0.021
Preoperative hemorrhage (verified by CT scan)	5.37	0.38–77.47	0.215
Preoperative leukocyte count > 12.5 Gpt/L	8.36	1.41–49.99	0.019

## 4 Discussion

This study investigates the impact of preoperative neurological complications, including both hemorrhagic and ischemic events, on the postoperative course and clinical outcomes in patients undergoing surgery for IE.

Neurological dysfunction is a common complication in IE, occurring in 10%–40% of cases [5]. This is similar to our results, where 20.9% of patients had a neurological event before surgery. These patients present a particular therapeutic challenge.

The previous 2015 version of the endocarditis guidelines recommended delaying surgical intervention for approximately 4 weeks in cases of intracranial hemorrhage. At the same time, patients who had experienced an ischemic stroke but presented with urgent surgical indications, such as severe heart failure, high embolic risk, or an uncontrolled infection, were advised to undergo surgery without delay. At that time, this recommendation had a class IIa indication [6].

The updated ESC guidelines of 2023 have further clarified these recommendations: For patients with ischemic stroke and strong surgical indications, immediate surgery is now clearly recommended (class I). In cases of hemorrhagic stroke, a more differentiated approach is now suggested. When bleeding is limited and the neurological condition is stable, early surgery may be considered. However, if the clinical situation allows, postponing surgical intervention for up to 4 weeks remains an option (class IIa) [4]. In the literature, contradictory data can be found. In 2024, Tsai et al. reported that patients with IE and neurological complications (both ischemic and hemorrhagic) who underwent surgery within the first 7 days had better outcomes than those with delayed surgery. Furthermore, in their subgroup analysis comparing patients with cerebral ischemia and those with cerebral hemorrhage, no significant differences were observed in in‐hospital or post‐discharge outcomes [7]. Similar results were reported by Zhang et al. [8]. On the other hand, several other authors suggested that it might be better to wait with surgery in some cases, such as after a major cerebral infarction or hemorrhagic complication [9, 10, 11]. Our data reflect the generally accepted recommendation that patients with neurologic complications should be evaluated carefully, which often results in a longer time span before surgery. The interval from diagnosis to surgery was longer in group B (with preoperative neurologic events) compared to group A (without) (22.5 vs. 33.2 days), and thereby also exceeded the waiting times reported in previous studies [12]. More specifically, patients with ischemic events or TIA (28/6 days) underwent surgery earlier, while those with cerebral hemorrhage had the longest waiting period (56 days), consistent with current guideline recommendations.

Consequently, preoperative neurological complications affect the postoperative course. That was reflected in our results. Patients in Group B showed a significantly longer duration of mechanical ventilation (63.7 vs. 59.3 h, *p* = 0.013), consistent with Wilbring et al. who reported 44 vs. 31 h (*p* = 0.05) [12].

Moreover, patients with preoperative hemorrhagic events had a notably increased risk of recurrent bleeding (OR 420.0, *p* < 0.001). This may be due to the full heparinization required during cardiopulmonary bypass. However, even with a waiting time of 33.2 days, as recommended by current guidelines [4] for patients in Group B, the risk of recurrent bleeding still remained high. This underlines the need for careful risk assessment by the endocarditis team to choose the best treatment strategy for each patient.

On the other hand, patients with embolic events were more likely to suffer postoperative embolism (OR 133.71, *p* < 0.001). Although the exact mechanism remains unclear, this finding is consistent with previous observations. Carrascal et al. also reported that postoperative cerebral events were often associated with prior embolic episodes [13].

After the hospital stay, the need for neurological rehabilitation was significantly higher in group B (8.8% vs. 25.6%, *p* = 0.008). In Germany, all patients with neurological symptoms are typically admitted to neurorehabilitation centers. Therefore, the fact that only 25.6% of patients of group B in our cohort required rehabilitation suggests that the most of them had either mild neurological deficits or showed neurological improvement during their hospital stay. Furthermore, our univariate logistic regression analysis showed that patients with preoperative cerebral bleeding had a 4.7 times higher risk of requiring neurological rehabilitation (*p* = 0.044).

Despite these complications, preoperative neurological deficits did not appear to influence overall mortality. Varela‐Barca et al. similarly found no predictive value of neurological deficits for mortality [14], and Carrascal et al. reported no significant difference in postoperative mortality between patients with and without preoperative neurological events (31.5% vs. 29.8%) [13].

Similarly, in our study, we also found no significant difference in 30‐day mortality between patients with and without preoperative neurological events (8.3% vs. 7.5%, *p* = 1.0). In contrast, Wilbring et al. reported a higher 30‐day mortality rate among patients with preoperative neurological events (20%) compared to those without (8.2%) [12].

Our study population included a high‐risk surgical cohort, with a mean predicted mortality risk of 33% based on EuroSCORE II. However, the actual 30‐day mortality was only 8.4%, showing excellent surgical outcomes at our center. Reported mortality rates in the literature range widely between 8% and 40% [15]. This favorable outcome is likely due to a well‐established multidisciplinary endocarditis team, comprising cardiology, microbiology, and cardiac surgery, as well as to the expertise of experienced surgeons performing the procedure.

On the other hand, EuroSCORE II may not be a reliable tool to estimate the true mortality risk. This limitation has also been addressed in recent literature [16]. For instance, Jusli‐Melcher et al. reported an in‐hospital mortality rate of approximately 17% in patients with a EuroSCORE II of 24.52 following surgery for infective endocarditis surgery [17], while Varela‐Barca et al. observed an even higher mortality rate of 26% in patients with a EuroSCORE II of just 14.1 [14].

Our multivariate logistic regression analysis identified preoperative leukocytosis, TIA, and embolism as independent risk factors for postoperative cerebral events. These findings are in agreement with previous research; Carrascal et al. also reported that preoperative neurological events, TIAs, embolisms, and leukocytosis significantly increased the risk of cerebral complications after IE surgery IE [13]. Similarly, Rossi et al. showed that IE patients who had neurological events before surgery were more likely to have new neurological complications after the operation [5].

Whether the type of pathogen influences patient outcomes remains an important question. Wilbring et al. reported a higher incidence of Staphylococcus infections in patients with neurological complications [12]. Similarly, Wang et al. found that survival outcomes depended on the causative pathogen [18]. We could not confirm this association in our cohort, as there were no significant differences in the incidence of infective organisms between the two groups. However, this non‐significant finding which differs from literature can be attributed to the small sample size, thus a type II error is most likely and might be absent in a larger cohort.

The effect of vegetation size on surgical outcomes in IE remains a matter of debate. While it is often questioned whether larger vegetations influence operative risk, the relationship between vegetation size and neurological complications is still unclear. Although the evidence is mixed, several studies, such as Nielsen 2024, suggest that combining surgery with antibiotic therapy may improve outcomes in patients with vegetations > 10 mm, particularly in preventing embolic events [19]. Wang et al. identified large vegetation size as an independent risk factor for neurological complications [20], and Jumah et al. reported that each millimeter increase in vegetation size was associated with a higher risk of acute ischemic stroke [21].

Despite the presence of larger vegetation sizes in Group B, our study demonstrated comparable operative duration and no significant difference in mortality. These findings align with those of Foc'sbol et al. which reported similar mortality rates in patients with vegetation > 10 mm and < 10 mm [22]. Furthermore, both our univariate logistic regression analysis and the subsequent multivariate model did not identify vegetation size as an independent risk factor for postoperative neurological complications. However, Carrascal et al. found that vegetations > 30 mm were predictive of increased postoperative mortality [13]. A major limitation of our study is the retrospective nature, the relatively small cohort size and, in particular, the very small subgroups when stratified by pathogen or type of complication, especially those with preoperative intracerebral hemorrhage. Only a few of these patients experienced recurrent bleeding after surgery, which led to an extremely high odds ratio (OR 420) with very wide confidence intervals. Clearly, such estimates are unstable and must be interpreted with caution. This also helps explain why preoperative hemorrhage did not emerge as an independent predictor in the multivariate analysis. To be fully transparent, we have provided the absolute numbers in the results section and tables. Overall, these findings highlight that the striking association seen in the univariate analysis is driven more by the low number of cases than by a robust statistical effect, and the study might be underpowered to show certain differences others have found in the literature in larger cohorts (e.g., more complications in *S. aureus* endocarditis).

Although a matter of ongoing debate, the retrospective design and limited sample size of our study do not allow for meaningful conclusions regarding the impact of early versus delayed surgery. Because no systematic comparison across different time windows was performed, our data cannot contribute to the ongoing debate on optimal surgical timing. Rather, our results should be interpreted as hypothesis‐generating, highlighting potential predictors of adverse outcomes rather than providing guidance on the timing of surgery.

## 5 Conclusion

Despite delayed operation interval after diagnosis, patients with preoperative neurological complications remain at a significantly higher risk for postoperative neurological deterioration. Our findings indicate that preoperative embolic or hemorrhagic events are strong predictors of prolonged mechanical ventilation, recurrent cerebrovascular complications, and an increased need for neurological rehabilitation. However, these factors did not significantly impact overall mortality of the patients. While vegetation size was not identified as an independent risk factor for new neurological events, preoperative leukocytosis, TIA, and embolism were associated with a higher risk of postoperative cerebral complications. These results underline the importance of carefully choosing the right timing for surgery and interdisciplinary management strategies to optimize outcomes in this high‐risk patient group.

## Conflicts of Interest

The authors declare no conflicts of interest.

## Supporting information


**TABLE S1:** Time between diagnosis and surgery (days) in patients of Group B.

## Data Availability

The data that support the findings of this study are available from the corresponding author upon reasonable request.
