# Supplementary material for: Infective Endocarditis—Impact of Preoperative Neurological Complications on Postoperative Outcome
Source: Eur J Neurol. 2026 Feb 19;33(2):e70539. doi: 10.1111/ene.70539 (PMC12920689; doi:10.1111/ene.70539)
Supplement: Supplementary file 1 — TABLE S1: Time between diagnosis and surgery (days) in patients of Group B. [file ENE-33-e70539-s001.docx]

***Supplementary table 1: Time between diagnosis and surgery (days) in patients of Group B***

| Group B: Preoperative neurologic event related to IE (stroke, embolism, hemorrhage, TIA) N=40 | | | | | |
| --- | --- | --- | --- | --- | --- |
|  | **Number of patients** | **Mean** | **SD** | **Median** | **Range (min–max)** |
| **Preoperative TIA** | 4 | 13.00 | 17.80 | 6.00 | 1–39 |
| **Preoperative cerebral ischemia** | 27 | 29.67 | 22.00 | 28.00 | 1–97 |
| **Preoperative cerebral hemorrhage** | 8 | 55.12 | 36.54 | 56.50 | 3–102 |
